# Knowledge, attitude, and behaviours concerning mpox from an online survey among MSM in the Eastern Mediterranean Region (EMR)

**DOI:** 10.1371/journal.pone.0360183

**Published:** 2026-11-13

**Authors:** Stephen J. McCall, Hazar Shamas, Mirna Bou Hamdan, Martine Najem, Ahmed S. Alaama, Muhammad Shahid Jamil, Jocelyn DeJong, Joumana Hermez, Nada M. Melhem

**Affiliations:** 1 Center for Research on Population and Health, Faculty of Health Sciences, American University of Beirut, Beirut, Lebanon; 2 Medical Laboratory Sciences Program, Division of Health Professions, Faculty of Health Sciences, American University of Beirut, Beirut, Lebanon; 3 Center for Public Health Practice, Faculty of Health Sciences, American University of Beirut, Beirut, Lebanon; 4 Department of Health Promotion, Disease Prevention and Control, World Health Organization Regional Office for the Eastern Mediterranean, Cairo, Egypt; 5 Department of Epidemiology and Population Health, Faculty of Health Sciences, American University of Beirut, Beirut, Lebanon; Virginia Commonwealth University, UNITED STATES OF AMERICA

## Abstract

**Background:**

Human monkeypox (mpox), a viral zoonotic infectious disease, was declared a public health emergency of international concern. Men who have sex with men (MSM) were most affected by mpox during the global outbreak. This study aimed to assess the knowledge, attitudes, and behaviours concerning mpox among the MSM population in the Eastern Mediterranean Region (EMR).

**Methods:**

This was a cross-sectional study of MSM residing in countries of the EMR. Participants were recruited through non-governmental organisations, dating applications, and LGBTQ+ websites between July and October 2023. Data on behaviours, perceptions, access to healthcare, socio-demographics, and medical characteristics related to mpox were collected online via LimeSurvey. The outcome was high mpox knowledge whereby a score greater than 12 of 17 was considered as high mpox knowledge score. Adaptive Least Absolute Shrinkage and Selection Operator logistic regression was used to predict participants’ knowledge about mpox. Model discrimination and calibration were assessed.

**Results:**

A total of 643 MSM participants of median age 31 (IQR:26–37) were included in the study. Among study participants, 131 (20.4%) had high mpox knowledge. Key predictors of high mpox knowledge score were being 31–40 years old, employed, in a relationship, thinking mpox vaccine is safe, and knowing someone infected with mpox. The model had good discrimination AUC = 0.812 (95%CI:0.77–0.85) and calibration C-slope = 1.073 (95%CI:0.87–1.27).

**Conclusion:**

This study identified gaps and predictors of high mpox knowledge score among MSM in the EMR. These findings provide new insights needed to raise awareness and inform targeted public health interventions to reduce the spread of mpox.

## Introduction

Human monkeypox (mpox) is a viral zoonotic infectious disease caused by the mpox virus (MPXV), a double-stranded DNA virus belonging to the Orthopoxvirus genus of the family Poxviridae [1]. MPXV consists of two distinct genetic clades: Congo Basin clade I, mainly in central Africa and specifically the Democratic Republic of the Congo and clade II, that was confined to West Africa prior to 2022 and has emerged globally in 33 lineages during the 2022–2024 outbreak [2–6]. In 2022, mpox was detected in 130 countries including non-endemic ones. Consequently, the World Health Organization (WHO) declared mpox as a public health emergency of international concern (PHEIC), the latter ended in May 2023. However, the emergence and spread of clade Ib led to reinstating a state of PHEIC in August 2024 [7]. As of March 10, 2025, 129,172 laboratory-confirmed cases and 283 deaths have been reported to WHO from 130 countries; 0.7% of those cases were reported in the Eastern Mediterranean Region (EMR) [8–10]. The first mpox case from the EMR was reported in the United Arab Emirates (UAE) on May 24, 2022 [8]. As of 10 March 2025, 886 confirmed mpox cases have been reported in the EMR [10].

Mpox is transmitted through close physical or sexual contact [3,9], with men who have sex with men (MSM) being the most affected population during the 2022–2024 global outbreak [7,8]. Between 2022 and 2024, 84.9% of the confirmed mpox cases were reported among MSM with genital and perianal rashes being the first symptoms of the disease [9–12]. Of the reported confirmed cases, 51.9% were people living with HIV (PLHIV), 10% had gonorrhoea, 4% had syphilis and < 1% had Herpes Simplex Virus [10,13,14]. In the EMR, 60% of the reported cases were among MSM and 10% among PLHIV [8]. Overall, the pooled hospitalisation rate caused by mpox is 10.5% (95% confidence interval:8.3–12.8) while case-fatality rates can reach up to 8.7%, with greater severity among immunocompromised individuals [2,3,10,13] with untreated and advanced HIV disease [14]. These findings underscore the importance of prioritising MSM, particularly those living with HIV, for mpox surveillance, prevention, early diagnosis and vaccination. Accordingly, on September 13, 2024, the strategic framework (2024–2027) declared the Strategic Advisory Group of Experts’ (SAGE) prequalification approval and recommendation for the use of the Bavarian Nordic A/S mpox vaccine (MVA-BN) for persons at high risk of exposure, including MSM [4,15].

Evidence from Europe and North America has highlighted important disparities in mpox-related knowledge, risk perception, and preventive behaviours among MSM populations. These studies have shown that mpox transmission has been closely linked to sexual networks and behaviours, often overlapping with other sexually transmitted infections, while stigma and discrimination remain key barriers to accessing testing, prevention, and care [16–18]. Moreover, novel community-based surveillance approaches using discarded condoms have also been proposed as a promising tool to enhance mpox and sexually transmitted infection (STI) monitoring in Global South settings where traditional surveillance infrastructure remains limited [19]. However, such evidence and surveillance approaches remain limited in the EMR, where sociocultural stigma and legal constraints may further hinder access to accurate information and services. Since stigma and social taboos significantly hinder the ability of MSM to access care, treatment, and prevention of sexually transmitted diseases, it remains important to understand the knowledge, attitudes, and practices (KAP) of MSM for early diagnosis, prevention, and timely management of mpox [7,20]. This is especially important with the emergence of new lineages of circulating clades [21,22]. Few studies examined KAP among MSM globally. The findings of these studies were inconsistent, whereby high mpox knowledge scores were reported in Australia, UK, and Brazil compared to low scores in Italy, China, Taiwan and Nepal [23–29].

The aim of this study was to assess knowledge, attitudes, and behaviours of the MSM population concerning mpox in the EMR.

## Materials and methods

### Study design and study population

This study was a cross-sectional survey among MSM. The study was reported using the Transparent Reporting of a Multivariable Prediction Model for Individual Prognosis or Diagnosis (TRIPOD) and Strengthening the Reporting of Observational Studies in Epidemiology (STROBE) guidelines. Individuals who were identified as MSM, ≥ 18 years old, and residing in countries of the EMR were included (S1 Appendix). Based on the definition of MSM by the Centers for Disease Control and Prevention, the study population included persons who are biologically male and had sexual relations with other men regardless of sexual orientation or gender identity. The study population also included transgender men and women based on whether men are defined by sex at birth (i.e., transgender women included) or current gender identity (i.e., transgender men included). The study was advertised in all 22 countries of the EMR: Afghanistan, Bahrain, Djibouti, Egypt, Islamic Republic of Iran, Iraq, Jordan, Kuwait, Lebanon, Libya, Morocco, Oman, Pakistan, Occupied Palestinian Territories, Qatar, Saudi Arabia, Somalia, Sudan, Syrian Arab Republic, Tunisia, UAE, and Yemen.

### Sampling design and data collection

Study subjects were recruited between July and October 2023. MSM were invited to participate through two modalities: (1) recruitment through non-governmental organisations (NGOs) offering services and providing counselling to MSM and (2) recruitment through advertisements on social media applications and websites. The first modality was through engagement with 12 NGOs offering health services and providing counselling to MSM in their respective countries (Tunisia, Morocco, Algeria, Pakistan, Jordan, Lebanon, and Egypt) (S1 Table). A QR code to the online link was shared with beneficiaries through different modalities including WhatsApp groups, Facebook, Instagram, SMS, emails, posters and flyers in healthcare centres of NGOs.

The second modality was through advertisements on lesbian, gay, bisexual, transgender, queer or questioning persons (LGBTQ+) dating applications and website platforms. A list of these platforms and websites is provided in supplementary material (S2 Table). A marketing company advertised the survey on gay dating applications and LGBTQ+ websites. Advertisements in the form of infographics were displayed for five weeks using Global Positioning System coordinates to target users within countries of the EMR. Participants from both modalities provided informed consent by selecting "Yes" on the form prior to starting the survey.

### Data collection tools

Data were collected online through LimeSurvey without including any identifiers. The survey was initially developed in English, then translated to Arabic and French. Data were collected in all three languages. Survey questions were developed based on previously published literature on knowledge, attitudes, and perceptions towards mpox as well as other infectious diseases [30–35]. The survey included questions based on the Integrated Behavioural Model (to assess attitude, perception and behavioural intentions) and the Precaution Adoption Process Model (to assess readiness to get vaccinated) [30,36,37]. Context specific questions were tailored to fit the EMR context. The survey consisted of the following sections: demographic characteristics, lifestyle behaviours, mpox knowledge, practices and behaviours, vaccination history, perception towards mpox, and STI history.

### Outcome

The main outcome of this study was high mpox knowledge. The section on mpox knowledge in the survey consisted of 17 questions. The knowledge items were scored as follows: zero if the answer was incorrect or “I don’t know” and one if the answer was correct. The maximum score a participant could get for mpox knowledge was 17. A higher knowledge score indicated more knowledge on mpox. Mpox knowledge was then dichotomised at the 75^th^ percentile of mpox knowledge score (score of >12 vs ≤ 12) with scores above 12 indicating high mpox knowledge.

### Candidate predictors

Candidate predictors were identified based on their presence in the literature and the study questionnaire. These predictors included: age, gender identity, marital status, employment status, education level, residence area, current smokers, alcohol use within the past 12 months, recreational drug use within the past 12 months, thinking the mpox vaccine is safe, knowing someone infected with mpox, willing to get mpox vaccine, willing to pay for mpox vaccine, HIV history, syphilis history, COVID-19 vaccine uptake, influenza vaccine uptake, and source of healthcare information.

### Statistical analysis

Data were cleaned and scrutinised for outliers and impossible values. Normality was checked for continuous variables using histograms. Descriptive analysis was performed including frequencies, percentages, median, and interquartile ranges (IQR). Bivariate logistic regression was conducted to assess the crude association between mpox knowledge score and the following characteristics: behaviours after learning about mpox, mpox perceptions, access to healthcare, socio-demographics, risky behaviour, and medical characteristics. A p-value <0.05 was considered for statistical significance.

Adaptive Least Absolute Shrinkage and Selection Operator (LASSO) was used to predict participants’ knowledge about mpox. All candidate predictors were incorporated for model development.

We further ran a multivariable logistic regression analysis on selected predictors to produce adjusted odds ratios (ORs) and 95% confidence intervals (CIs). The final model’s discrimination using the Area Under the Receiver Operating Characteristic Curve (AUC) and calibration using the calibration slope (C-slope) were assessed as measures of model performance. Statistical analysis was performed using Stata version 18.

### Ethical approval and reporting

The study proposal, questionnaire, advertisements, and consent forms were approved by the Institutional Review Board (IRB) at the American University of Beirut (AUB) (reference SBS-2022–0347) and cleared by the WHO ethical review committee of the Eastern Mediterranean Region.

## Results

### Demographic, medical, and risky behaviours among study participants

The online survey was accessed by a total of 1,643 participants between July and October 2023 using both modalities, out of which 643 MSM participants completed the survey and were eligible to be included in the study (S1 Fig). Participants resided in 15 out of the 22 EMR countries with Morocco accounting for the highest proportion of participants (30.9%). The median age of study participants (n = 643) was 31 (IQR: 26–37). Almost all study participants identified their biological sex as males (98.8%) while around 0.9% were of female biological sex but considered MSM due to identifying themselves as gender fluid or transgender and had sex with at least 1 man in the past year. Moreover, 0.3% of the study participants included those who preferred not to disclose their biological sex but belonged to the MSM community since they identified themselves as males and had sex with at least 1 man in the past year. Almost half of the study participants had a university or college degree (47.7%), and were employed (55.8%) (Table 1).

**Table 1:** Demographic, medical, and risky behaviour characteristics among study participants.

	n	(%)
**Total Participants**	643	(100)
**SOCIO-DEMOGRAPHICS**		
**Age**		
18–30	308	(47.9)
31–40	240	(37.3)
41–50	82	(12.7)
More than 50	13	(2.1)
**Country of residence**		
Morocco	199	(30.9)
Egypt	169	(26.3)
Lebanon	97	(15.1)
Tunisia	62	(9.6)
United Arab Emirates	37	(5.8)
Pakistan	20	(3.1)
Kingdom of Saudi Arabia	18	(2.8)
Jordan	15	(2.3)
Other EMR countries**	23	(3.6)
Prefer not to say	3	(0.5)
**Sex at birth**		
Male	635	(98.8)
Female	6	(0.9)
Prefer not to say	2	(0.3)
**Gender identity**		
Male	472	(73.4)
Trans (transgender)	95	(14.8)
Gender fluid	36	(5.6)
Female	12	(1.9)
Other	5	(0.8)
Prefer not to say	23	(3.6)
**Marital status**		
Single	392	(61.0)
Relationship	104	(16.2)
Married to a woman	50	(7.8)
Married to a man	11	(1.7)
Prefer not to say	86	(13.3)
**Education level**		
Never attended school	5	(0.8)
Primary school	24	(3.7)
Middle school	37	(5.8)
High school	98	(15.2)
Technical vocational school	47	(7.3)
University/college	307	(47.7)
Postgraduate	117	(18.2)
Prefer not to say	8	(1.2)
**Employment status**		
Student	99	(15.4)
Employed	359	(55.8)
Unemployed	122	(19.0)
Other	30	(4.7)
Prefer not to say	33	(5.1)
**MPOX TESTING HISTORY**		
**Ever had a test for mpox**		
No	596	(92.7)
Yes	13	(2.0)
Prefer not to say	34	(5.3)
**VACCINATION**		
**COVID-19 vaccine history**		
No	65	(10.1)
Yes	559	(86.9)
Prefer not to say	19	(3.0)
**Influenza vaccine history**		
No	337	(52.4)
Yes	180	(28.0)
I don’t remember	93	(14.5)
Prefer not to say	33	(5.1)
**STI and VIRAL HEPATITIS HISTORY**		
**STI testing history**		
No	74	(11.5)
Yes	504	(78.4)
I don’t know	20	(3.1)
Prefer not to say	45	(7.0)
**HIV status**		
Negative	313	(48.7)
Positive	244	(37.9)
I don’t know	19	(3.0)
Prefer not to say	67	(10.4)
**History of hepatitis B**		
Negative	431	(67.0)
Positive	103	(16.0)
I don’t know	36	(5.6)
Prefer not to say	73	(11.4)
**History of hepatitis C**		
Negative	440	(68.4)
Positive	93	(14.5)
I don’t know	35	(5.4)
Prefer not to say	75	(11.7)
**History of syphilis**		
Negative	398	(61.9)
Positive	127	(19.8)
I don’t know	39	(6.1)
Prefer not to say	79	(12.3)
**History of chlamydia**		
Negative	437	(68.0)
Positive	72	(11.2)
I don’t know	56	(8.7)
Prefer not to say	78	(12.1)
**History of gonorrhoea**		
Negative	365	(56.8)
Positive	148	(23.0)
I don’t know	46	(7.2)
Prefer not to say	84	(13.1)
**Total history of STIs and viral hepatitis**		
No STI or viral hepatitis	107	(16.6)
1 STI or viral hepatitis	207	(32.2)
2 STIs or viral hepatitis	145	(22.6)
3 STIs or viral hepatitis	68	(10.6)
4 or more STIs or viral hepatitis	116	(18.0)

**Other EMR countries: Countries with 10 or less participants; Bahrain, Iran, Iraq, Kuwait, Qatar, Sudan, Yemen.

The majority of study participants had not been tested before for mpox (92.7%). Results showed that 38% had a history of HIV infection, followed by 23.0% of gonorrhoea, 19.8% of syphilis, and 16.0% of hepatitis B virus (HBV) among others. Results also showed that 22.6% and 18.0% of participants had a history of 2 and 4 or more STIs/ viral hepatitis, respectively (Table 1).

### Mpox knowledge in relation to socio-demographic and STI history of study participants

The association between the socio-demographic and clinical characteristics of the study participants and their knowledge about mpox was assessed (Table 2). Approximately, 20% of the study participants had a high knowledge score (mpox knowledge score >12). Participants who identified their gender as other than males or females had higher odds of high mpox knowledge score compared to those who identified themselves as males (OR:2.03; 95%CI:1.31–3.13). Factors associated with higher mpox knowledge also included attending postgraduate school compared to not attending school or attending primary school only (OR:5.30; 95%CI:1.19–23.5), being employed compared to being a student (OR:4.24; 95%CI:2.06–8.73), being in a relationship compared to being single (OR:5.13; 95%CI:3.07–8.57), and knowing someone infected with mpox compared to not knowing someone infected with mpox (OR:6.43; 95%CI:4.20–9.85).

**Table 2:** Mpox knowledge among study participants.

	Total		Low (score≤12)		High (score>12)		OR	(95%CI)
	n	(%)	n = 512	(79.63%)	n = 131	(20.37%)		
**SOCIO-DEMOGRAPHICS**								
**Age**								
18–30	308	(47.90)	274	(53.50)	34	(26.00)	1	
31–40	240	(37.30)	164	(32.00)	76	(58.00)	**3.73**	**(2.38–5.84)**
41–50	82	(12.75)	64	(12.50)	18	(13.70)	**2.26**	**(1.20–4.26)**
More than 50	13	(2.05)	10	(2.00)	3	(2.30)	2.41	(0.63–9.21)
**Gender identity**								
Male	472	(73.40)	387	(75.60)	85	(64.90)	1	
Female	12	(1.90)	10	(2.00)	2	(1.50)	0.91	(0.19–4.23)
Other	136	(21.20)	94	(18.40)	42	(32.10)	**2.03**	**(1.31–3.13)**
Prefer not to say	23	(3.60)	21	(4.10)	2	(1.50)	0.43	(0.09–1.88)
**Education level**								
Never attended school and primary school	29	(4.50)	27	(5.30)	2	(1.50)	1	
Middle and high school	135	(21.00)	124	(24.20)	11	(8.40)	1.19	(0.25–5.71)
Technical school and college	354	(55.00)	272	(53.10)	82	(62.60)	4.06	(0.94–17.40)
Postgraduate school	117	(18.20)	84	(16.40)	33	(25.20)	**5.30**	**(1.19–23.50)**
Prefer not to say	8	(1.20)	5	(1.00)	3	(2.30)	**8.09**	**(1.06–61.50)**
**Employment status**								
Student	99	(15.40)	90	(17.60)	9	(6.90)	1	
Unemployed	122	(18.97)	113	(22.10)	9	(6.90)	0.79	(0.30–2.08)
Employed	359	(55.83)	252	(49.20)	107	(81.70)	**4.24**	**(2.06–8.73)**
Other	30	(4.67)	28	(5.47)	2	(1.53)	0.71	(0.14–3.50)
Prefer not to say	33	(5.13)	29	(5.70)	4	(3.10)	1.37	(0.39–4.81)
**Marital status**								
Single	392	(60.96)	351	(68.60)	41	(31.30)	1	
Relationship	104	(16.17)	65	(12.70)	39	(29.80)	**5.13**	**(3.07–8.57)**
Married	61	(9.49)	50	(9.80)	11	(8.40)	1.88	(0.90–3.90)
Prefer not to say	86	(13.37)	46	(9.00)	40	(30.50)	**7.44**	**(4.36–12.68)**
**MPOX TESTING HISTORY**								
**Ever had a test for mpox**								
No	596	(92.70)	483	(94.30)	113	(86.30)	1	
Yes	13	(2.00)	8	(1.60)	5	(3.80)	2.67	(0.85–8.31)
Prefer not to say	34	(5.30)	21	(4.10)	13	(9.90)	**2.64**	**(1.28–5.44)**
**Know someone infected with mpox**								
No	480	(74.65)	420	(82.00)	60	(45.80)	1	
Yes	142	(22.08)	74	(14.50)	68	(51.90)	**6.43**	**(4.20–9.85)**
Prefer not to say	21	(3.27)	18	(3.50)	3	(2.30)	1.17	(0.33–4.08)
**STI and VIRAL HEPATITIS HISTORY**								
**HIV status**								
Negative	313	(48.70)	249	(48.60)	64	(48.90)	1	
Positive	244	(37.90)	202	(39.50)	42	(32.10)	0.81	(0.52–1.24)
Prefer not to say	86	(13.40)	61	(11.90)	25	(19.10)	1.59	(0.92–2.73)
**History of hepatitis B**								
Negative	540	(84.00)	427	(83.40)	113	(86.30)	1	
Positive	103	(16.00)	85	(16.60)	18	(13.70)	0.8	(0.46–1.38)
**History of hepatitis C**								
Negative	550	(85.50)	432	(84.40)	118	(90.10)	1	
Positive	93	(14.50)	80	(15.60)	13	(9.90)	0.59	(0.31–1.10)
**History of syphilis**								
Negative	398	(61.90)	332	(64.80)	66	(50.40)	1	
Positive	127	(19.80)	111	(21.70)	16	(12.20)	0.72	(0.40–1.30)
Prefer not to say	118	(18.40)	69	(13.50)	49	(37.40)	**3.57**	**(2.27–5.61)**
**History of chlamydia**								
Negative	571	(88.80)	459	(89.60)	112	(85.50)	1	
Positive	72	(11.20)	53	(10.40)	19	(14.50)	1.46	(0.83–2.58)
**History of gonorrhoea**								
Negative	495	(77.00)	387	(75.60)	108	(82.40)	1	
Positive	148	(23.00)	125	(24.40)	23	(17.60)	0.65	(0.40–1.07)
**Total history of STIs and viral hepatitis**								
No STI or viral hepatitis	107	(16.60)	84	(16.40)	23	(17.60)	1	
1 STI or viral hepatitis	207	(32.20)	154	(30.10)	53	(40.50)	1.25	(0.72–2.19)
2 STIs or viral hepatitis	145	(22.60)	121	(23.60)	24	(18.30)	0.72	(0.38–1.36)
3 STIs or viral hepatitis	68	(10.60)	56	(10.90)	12	(9.20)	0.78	(0.36–1.69)
4 or more STIs or viral hepatitis	116	(18.00)	97	(18.90)	19	(14.50)	0.71	(0.36–1.40)

### Mpox knowledge in relation to risky behaviours and practices of study participants

We assessed knowledge in relation to risky behaviours and practices among study participants (Table 3). Results showed that participants who reported reducing their risky behaviours after learning about mpox (including the number of sex partners, involvement in group sex, the number of casual partners or one-time encounters, or attendance at sex parties) were more likely to have high mpox knowledge compared to those who did not.

**Table 3:** Risky practices, behaviour, and mpox knowledge among study participants.

	Total		Low (score≤12)		High (score>12)		OR	(95%CI)
	n	(%)	n = 512	(79.63%)	n = 131	(20.37%)		
**Since learning about mpox, how have any of the following changed**								
**Number of sex partners**								
Not changed	236	(36.70)	192	(37.50)	44	(33.60)	1	
Decreased	266	(41.40)	187	(36.50)	79	(60.30)	**1.84**	**(1.21–2.80)**
Increased	18	(2.80)	15	(2.90)	3	(2.30)	0.87	(0.24–3.14)
Not applicable	63	(9.80)	60	(11.70)	3	(2.30)	**0.21**	**(0.06–0.72)**
Prefer not to say	60	(9.30)	58	(11.30)	2	(1.50)	**0.15**	**(0.03–0.63)**
**Having group sex**								
Not changed	143	(22.20)	112	(21.90)	31	(23.70)	1	
Decreased	223	(34.70)	154	(30.10)	69	(52.70)	1.62	(0.99–2.63)
Increased	35	(5.40)	23	(4.50)	12	(9.20)	1.88	(0.84–4.20)
Not applicable	191	(29.70)	175	(34.20)	16	(12.20)	**0.33**	**(0.17–0.63)**
Prefer not to say	51	(7.90)	48	(9.40)	3	(2.30)	**0.22**	**(0.06–0.77)**
**Use of condoms**								
Not changed	203	(31.60)	168	(32.80)	35	(26.70)	1	
Decreased	81	(12.60)	47	(9.20)	34	(26.00)	**3.43**	**(1.95–6.15)**
Increased	266	(41.40)	213	(41.60)	53	(40.50)	1.19	(0.74–1.91)
Not applicable	55	(8.60)	49	(9.60)	6	(4.60)	0.58	(0.23–1.47)
Prefer not to say	38	(5.90)	35	(6.80)	3	(2.30)	0.41	(0.12–1.41)
**Sex with casual partner/ one-time sexual encounter**								
Not changed	208	(32.30)	168	(32.80)	40	(30.50)	1	
Decreased	229	(35.60)	162	(31.60)	67	(51.10)	**1.73**	**(1.11–2.71)**
Increased	66	(10.30)	54	(10.50)	12	(9.20)	0.93	(0.45–1.90)
Not applicable	88	(13.70)	79	(15.40)	9	(6.90)	0.47	(0.22–1.03)
Prefer not to say	52	(8.10)	49	(9.60)	3	(2.30)	**0.25**	**(0.07–0.86)**
**Wore clothing during sex to reduce skin to skin contact**								
Not changed	221	(34.40)	181	(35.40)	40	(30.50)	1	
Decreased	112	(17.40)	66	(12.90)	46	(35.10)	**3.15**	**(1.89–5.24)**
Increased	111	(17.30)	81	(15.80)	30	(22.90)	1.67	(0.97–2.87)
Not applicable	139	(21.60)	128	(25.00)	11	(8.40)	**0.38**	**(0.19–0.78)**
Prefer not to say	60	(9.30)	56	(10.90)	4	(3.10)	**0.32**	**(0.11–0.94)**
**Going to sex venues or events**								
Not changed	159	(24.70)	125	(24.40)	34	(26.00)	1	
Decreased	193	(30.00)	137	(26.80)	56	(42.70)	1.50	(0.92–2.43)
Increased	60	(9.30)	40	(7.80)	20	(15.30)	1.83	(0.95–3.54)
Not applicable	186	(28.90)	167	(32.60)	19	(14.50)	**0.41**	**(0.22–0.76)**
Prefer not to say	45	(7.00)	43	(8.40)	2	(1.50)	**0.17**	**(0.03–0.74)**
Ever injected drugs in the past 12 months (n = 33)*								
No	3	(9.10)	3	(10.00)	0	(0.00)	–	–
Yes	30	(90.90)	27	(90.00)	3	(100.00)	–	–
**Able to abstain from any sexual activity, if you tested positive for mpox**								
No	95	(14.80)	71	(13.90)	24	(18.30)	1	
Yes	477	(74.20)	376	(73.40)	101	(77.10)	0.79	(0.47–1.32)
I don’t know	57	(8.90)	51	(10.00)	6	(4.60)	**0.34**	**(0.13–0.91)**
Prefer not to say	14	(2.20)	14	(2.70)	0	(0.00)	–	–
**Able to abstain from any sexual activity if you have been in contact with someone with mpox**								
No	120	(18.70)	85	(16.60)	35	(26.70)	1	
Yes	440	(68.40)	356	(69.50)	84	(64.10)	**0.57**	**(0.36–0.91)**
I don’t know	69	(10.70)	58	(11.30)	11	(8.40)	**0.46**	**(0.21–0.98)**
Prefer not to say	14	(2.20)	13	(2.50)	1	(0.80)	0.18	(0.02–1.48)
**Inform your sexual partner(s) if tested positive for mpox**								
No	120	(18.70)	86	(16.80)	34	(26.00)	1	
Yes	432	(67.20)	348	(68.00)	84	(64.10)	**0.61**	**(0.38–0.97)**
I don’t know	77	(12.00)	65	(12.70)	12	(9.20)	**0.46**	**(0.22–0.97)**
Prefer not to say	14	(2.20)	13	(2.50)	1	(0.80)	0.19	(0.02–1.54)
**Inform sexual partner(s) if you have been in contact with someone with mpox**								
No	155	(24.10)	120	(23.40)	35	(26.70)	1	
Yes	387	(60.20)	306	(59.80)	81	(61.80)	0.91	(0.57–1.42)
I don’t know	81	(12.60)	66	(12.90)	15	(11.50)	0.77	(0.39–1.53)
Prefer not to say	20	(3.10)	20	(3.90)	0	(0.00)	–	–
**Willing to share details of last sexual encounter**								
No	161	(25.00)	121	(23.60)	40	(30.50)	1	
Yes	300	(46.70)	257	(50.20)	43	(32.80)	**0.51**	**(0.31–0.81)**
I don’t know	147	(22.90)	102	(19.90)	45	(34.40)	1.33	(0.81–2.21)
Prefer not to say	35	(5.40)	32	(6.20)	3	(2.30)	**0.28**	**(0.08–0.97)**

*Only participants who answered “Yes” to “Have you ever injected drugs?” in the study questionnaire were able to respond to this question.

### Mpox knowledge in relation to perception and access to care among study participants

Knowledge in relation to participants’ perception of mpox and their access to care was also assessed (Table 4). Results showed that participants who perceived that they “are susceptible to mpox” (OR:2.44; 95%CI:1.42–4.16), and that “mpox vaccine is safe” (OR:2.37; 95%CI:1.34–4.21) had higher odds of high mpox knowledge compared to those who did not (Table 4). Moreover, 50.7% of the participants did not know where to get tested for mpox (S3 Table).

**Table 4:** Mpox perceptions and access to care by mpox knowledge among study participants.

	Total		Low (score≤12)		High (score>12)		OR	(95%CI)
	n	(%)	n = 512	(79.63%)	n = 131	(20.37%)		
**Vaccines are safe and effective**								
No	113	(17.60)	99	(19.30)	14	(10.70)	1	
Yes	434	(67.50)	323	(63.10)	111	(84.70)	**2.43**	**(1.33–4.42)**
I don’t know	96	(14.90)	90	(17.60)	6	(4.60)	0.47	(0.17–1.27)
**Mpox vaccine is safe**								
No	101	(15.70)	84	(16.40)	17	(13.00)	1	
Yes	314	(48.80)	212	(41.40)	102	(77.90)	**2.37**	**(1.34–4.21)**
I don’t know	228	(35.50)	216	(42.20)	12	(9.20)	**0.27**	**(0.12–0.59)**
**I consider myself susceptible to mpox**								
Disagree	165	(25.70)	144	(28.10)	21	(16.00)	1	
Neutral	183	(28.50)	144	(28.10)	39	(29.80)	**1.85**	**(1.04–3.31)**
Agree	259	(40.30)	191	(37.30)	68	(51.90)	**2.44**	**(1.42–4.16)**
Prefer not to say	36	(5.60)	33	(6.40)	3	(2.30)	0.62	(0.17–2.21)
**Mpox is dangerous and lethal**								
Disagree	89	(13.80)	73	(14.30)	16	(12.20)	1	
Neutral	169	(26.30)	150	(29.30)	19	(14.50)	0.57	(0.28–1.18)
Agree	345	(53.70)	252	(49.20)	93	(71.00)	1.68	(0.93–3.04)
Prefer not to say	40	(6.20)	37	(7.20)	3	(2.30)	0.36	(0.10–1.35)
**Mpox is easily preventable and controlled**								
Disagree	80	(12.40)	68	(13.30)	12	(9.20)	1	
Neutral	181	(28.10)	164	(32.00)	17	(13.00)	0.59	(0.27–1.30)
Agree	349	(54.30)	248	(48.40)	101	(77.10)	**2.31**	**(1.20–4.45)**
Prefer not to say	33	(5.10)	32	(6.20)	1	(0.80)	0.18	(0.02–1.42)
**Mpox is a laboratory made virus**								
Disagree	166	(25.80)	137	(26.80)	29	(22.10)	1	
Neutral	214	(33.30)	190	(37.10)	24	(18.30)	0.60	(0.33–1.07)
Agree	209	(32.50)	134	(26.20)	75	(57.30)	**2.64**	**(1.62–4.32)**
Prefer not to say	54	(8.40)	51	(10.00)	3	(2.30)	**0.28**	**(0.08–0.95)**
**Mpox is an attempt to reduce the population size**								
Disagree	188	(29.20)	154	(30.10)	34	(26.00)	1	
Neutral	198	(30.80)	172	(33.60)	26	(19.80)	0.68	(0.39–1.19)
Agree	199	(30.90)	137	(26.80)	62	(47.30)	**2.04**	**(1.27–3.31)**
Prefer not to say	58	(9.00)	49	(9.60)	9	(6.90)	0.83	(0.37–1.85)
**Pharmaceutical companies create epidemics to increase their profits**								
Disagree	167	(26.00)	127	(24.80)	40	(30.50)	1	
Neutral	205	(31.90)	178	(34.80)	27	(20.60)	**0.48**	**(0.28–0.82)**
Agree	208	(32.30)	152	(29.70)	56	(42.70)	1.16	(0.73–1.87)
Prefer not to say	63	(9.80)	55	(10.70)	8	(6.10)	0.46	(0.20–1.05)
**Mainstream media propagate unnecessary fear**								
Disagree	158	(24.60)	122	(23.80)	36	(27.50)	1	
Neutral	210	(32.70)	181	(35.40)	29	(22.10)	**0.54**	**(0.31–0.93)**
Agree	218	(33.90)	162	(31.60)	56	(42.70)	1.17	(0.72–1.89)
Prefer not to say	57	(8.90)	47	(9.20)	10	(7.60)	0.72	(0.33–1.56)

### Predictors of mpox knowledge among study participants

Predictors of high mpox knowledge included: age, gender identity, marital status, employment status, thinking mpox vaccine is safe, and knowing someone infected with mpox (Table 5). Accordingly, participants 31–40 years old had higher odds of high mpox knowledge compared to those aged between 18 and 30 years old. Moreover, participants who were employed, in a relationship, thought the vaccine is safe, or knew someone infected with mpox had higher odds of high mpox knowledge compared to those who did not. This model had good discrimination with AUC = 0.812 (95%CI:0.77–0.85), i.e., the model can accurately discriminate between those of higher and lower mpox knowledge score. The model had good calibration of C-slope = 1.073 (95%CI:0.87–1.27) (S2 Fig). Results from the multivariable logistic regression analysis for selected predictors are presented in Table 5.

**Table 5:** Predictors for high mpox knowledge.

Predictors	Coefficient	aOR	(95%CI)
**Age**			
18-30	−0.238371	1	
31-40	0.3830969	**2.17**	**(1.25–3.79)**
41-50	–	1.37	(0.63–2.94)
More than 50	–	4.34	(0.96–19.56)
**Gender identity**			
Male	–	1	
Female	–	0.89	(0.16–4.81)
Other	–	0.85	(0.48–1.50)
Prefer not to say	−1.020848	**0.16**	**(0.03–0.85)**
**Marital status**			
Single	−0.2680982	1	
Relationship	0.9467178	**3.89**	**(2.10–7.20)**
Married	–	1.22	(0.55–2.69)
Prefer not to say	0.9002329	**3.74**	**(1.90–7.36)**
**Employment status**			
Student	–	1	
Unemployed	–	0.64	(0.22–1.81)
Employed	0.6453472	1.33	(0.57–3.09)
Retired	–	–	–
Other	–	0.49	(0.08–2.71)
Prefer not to say	–	0.73	(0.18–2.91)
**Mpox vaccine is safe**			
No	–	1	
Yes	1.001069	**2.44**	**(1.22–4.87)**
I don’t know	–	0.61	(0.20–1.85)
**Know someone infected with mpox**			
No	–	1	
Yes	1.13184	**3.06**	**(1.81–5.18)**
Prefer not to say		1.01	(0.24–4.11)
**Intercept**	−3.15499		

Coefficients are from the adaptive LASSO; ‘-’ indicates the level was not selected or aOR did not converge; aORs are results from the multivariable logistic regression that included all selected predictors.

## Discussion

This study is unique in terms of MSM recruitment through NGOs and dating applications in the EMR region to assess the knowledge, attitudes, and behaviours related to mpox. Participants who reduced risky behaviours after learning about mpox or perceived themselves as susceptible to infection had higher mpox knowlegde. Older age, being in a relationship, being employed, thinking mpox vaccine is safe, and knowing someone infected with mpox were predictors of higher knowledge scores. A model predicting mpox knowledge score was developed with good discrimination and calibration.

Our data showed that almost 20% of the study participants had high mpox knowledge score; the latter is lower than the overall prevalence of good mpox knowledge worldwide (33%) [31,32]. Our results are in agreement with previous data reporting low knowledge scores among MSM in Italy, China, Taiwan and Nepal [24,26,27,29]. However, our data contrasts with previous studies reporting high mpox knowledge scores among MSM in Australia, UK, and Brazil [23,25,28].

In the EMR, mpox knowledge was mainly assessed among the general population, university students and healthcare professionals with inconsistent findings [11,35,38–47]. Good mpox knowledge was reported among university students in UAE and the general population in the Kingdom of Saudi Arabia (KSA) [39,41] while a moderate mpox knowledge was reported among the general population in Iraq [38]. Poor mpox knowledge was reported among medical students in KSA and healthcare professionals in Lebanon [11,40].

These variations in reporting along with relatively lower levels of knowledge observed in our study, may reflect underlying structural and contextual challenges within the region. Our findings reflect broader structural and health system challenges in the EMR that may affect mpox diagnosis to post-diagnosis care. Limited awareness and knowledge gaps identified in this study may contribute to delayed recognition of symptoms and reduced health seeking behaviour [16,18]. Moreover, stigma surrounding same-sex behaviour, compounded by legal and sociocultural constraints, may discourage individuals from seeking timely testing, disclosing symptoms, or engaging with healthcare services. These barriers may contribute to underdiagnosis and ongoing transmission within affected communities.

Our results showed an association between educational levels, age, being in a relationship, and having a history of STI with high mpox knowledge. These results are in agreement with data reported among MSM in China and Taiwan [26,27]. Patients with STIs, especially those with HIV infection, might have access to information related to transmission, symptoms and preventions of various STIs including mpox [26]. Older age and education were also associated with higher mpox knowledge among medical students in KSA [11]. Factors associated with mpox knowledge among the general Iraqi population included age, gender, marital status, and level of education [38]. Education may promote participation in health educational programmes addressing transmission of MPXV and preventive measures [35,48–50]. MSM who are 31–40 years had higher odds of high mpox knowledge as compared to younger ages. This was in agreement with data reported among university students in the UAE and among the general MSM population in China [26,39].

Although most evidence is derived from relatively educated populations, data suggest that individuals with lower educational attainment in the general population may be at increased risk of limited mpox knowledge. This highlights the need for public health strategies that extend beyond university settings to reach more diverse populations, particularly those with lower educational backgrounds. Addressing the knowledge gaps among young age groups through enhanced awareness is important to prevent and control the spread of mpox.

Our study identified employment as a predictor of high mpox knowledge, consistent with findings from KSA, where being employed and living in a country with recent mpox cases were both linked to greater knowledge [35]. Employed people may have greater access to different healthcare services and thus better information. Our results are also in agreement with data reported in KSA [41] and China [48] whereby knowledge of someone with mpox was associated with higher mpox knowledge. Participants who know someone with mpox or reside in countries with reported mpox cases might have higher awareness of the risks associated with disease pathogenicity and modes of transmission [26,49]. Moreover, knowing someone infected with mpox could predict higher mpox knowledge, potentially through increased health-seeking behaviours and health awareness [26,50].

Our results showed that risky behaviours declined following learning about mpox and were associated with high mpox knowledge. This finding is similar to data reported among MSM and students in the United States (US), gay, bisexual or queer participants (GBQ) in Australia, and gay, bisexual, and other MSM in the UK [28,49,51,52]. Similarly, a study among MSM in China reported the association between high mpox knowledge and prevention self-efficacy and reduction in risky behaviours [53]. Scarce data exist in the region on the above whereby one study originating from the KSA reporting willingness to comply with preventive measures and reduce risky behaviours after learning about mpox [54]. Reduction in risky behaviours could be driven by perceived susceptibility towards infection with MPXV and concern about contracting mpox after learning about it; thus, it remains essential to make available health information for those at higher risk of mpox [27,49]. In the EMR, the association of risky behaviours and mpox knowledge is understudied and requires further investigation [11,47,55,56].

This study showed that MSM who perceive themselves to be susceptible to mpox have higher mpox knowledge. This study also reported participants who trust that the mpox vaccine is safe reported higher mpox knowledge. These findings are consistent with studies conducted among the MSM community in the US and the WHO European Region, healthcare workers in Vietnam, GBQ in Australia, and students in China [49,57–59].

In the EMR, perceptions towards mpox were studied among the general population, medical students, and healthcare workers [11,35,40,47,55]. Results of those studies were similar to this study where participants who perceived themselves as susceptible to mpox had higher mpox knowledge [35,40,47,55,56]. Our results were also similar to studies conducted among the general population in Jordan, Yemen, UAE, and KSA [35,41,47,55,56]. Studies whose participants perceive mpox vaccine is safe learnt about mpox through awareness campaigns, MSM social media channels, and information spread on social media campaigns [55,59–61]. These findings highlight the importance of accessible and targeted communication strategies to improve mpox knowledge and promote mpox vaccination among MSM.

This study revealed knowledge gaps about mpox among MSM in the region. Countries of the region are urged to commit the necessary efforts and resources to prevent and control the spread of mpox through the development and implementation of a comprehensive plan. This comprehensive and strategic plan should include: a) capitalising on existing national health programmes and the integration of mpox into existing HIV and STI prevention programmes; this is important specifically for vulnerable groups to ensure equitable access to care; b) the alignment of the role and functions of NGOs providing services to high-risk groups with other national healthcare providers including but not limited to awareness campaigns, addressing misconceptions as well as knowledge gaps about mpox; c) ensuring the availability and access to diagnostic testing and therapeutics; d) promoting vaccination programmes and campaigns in addition to ensuring the availability of vaccines ready for rapid deployment; e) enhancing research in the region on MPXV transmission dynamics, risk factors, use and effectiveness of mpox vaccines, viral evolution and genomic sequencing and most importantly transparent data sharing. These recommendations showcase the need for continuous intersectoral coordination for scalable and equitable access to knowledge and care.

This study has several strengths. To our knowledge, it is among the first studies in the EMR to assess mpox-related knowledge, attitudes, and behaviours specifically among MSM recruited through both NGOs and dating applications; thus allowing access to a population, often difficult to reach, through conventional sampling approaches. The inclusion of multiple recruitment modalities also enabled the study to capture participants with different levels of engagement with community-based services.

Despite these strengths, this study had few limitations. The study used a convenience sampling approach where participants recruited through NGOs may have had access to awareness and educational materials. However, LGBTQ+ populations are often hidden in the EMR region due to fear of discrimination and stigma. We also acknowledge the lack of comprehensive data on treatment outcome and vaccine availability in the Middle East and North Africa region which might have affected our results. Moreover, a substantial proportion of participants selected “Prefer not to say” for several sociodemographic and behavioural variables. The reasons for the latter may be related to concerns regarding privacy, stigma, or the nature of the questions.

## Conclusions

In the context of the EMR and hard-to-reach populations, it remains essential to identify viable modalities to improve knowledge about health conditions that impact the MSM community in order to reduce risky behaviours and improve access to care. Addressing knowledge challenges requires culturally sensitive and inclusive public health approaches, including community engagement with trusted organisations and the provision of confidential, non-discriminatory healthcare services. This study highlights that those with higher knowledge of mpox had reduced risky behaviour after learning about mpox; it also identified the demographics of populations with a low mpox knowledge score to allow for health promotion targeting.

## Supporting information

S1 Appendix Study questionnaire. (DOCX)

S1 Table List of the NGOs that aided in data collection. (DOCX)

S2 Table List of the dating applications and LGBTQ+ websites the mpox survey was advertised on. (DOCX)

S3 Table Access to healthcare services and stigma related to mpox by knowledge among study participants. (DOCX)

S1 Fig Flow diagram of study participants selection. (DOCX)

S2 Fig LASSO Adaptive optimised-adjusted model performance. (DOCX)
