# Supplementary material for: Knowledge, attitude, and behaviours concerning mpox from an online survey among MSM in the Eastern Mediterranean Region (EMR)
Source: PLoS One. 2026 Nov 13;21(11):e0360183. doi: 10.1371/journal.pone.0360183 (PMC13766248; doi:10.1371/journal.pone.0360183)
Supplement: Supplementary file 1 [file pone.0360183.s001.docx]

# Methods:

EMR countries:  Bahrain, Kuwait, Oman, Qatar, Saudi Arabia, United Arab Emirates, Egypt, Iran, Iraq, Jordan, Lebanon, Libya, Morocco, Tunisia, occupied Palestinian territories (West Bank and Gaza Strip), Djibouti, Afghanistan, Pakistan, Somalia, Sudan, Yemen, Syrian Arab Republic

**S1 Appendix. Study Questionnaire**
