# Supplementary material for: Knowledge, attitude, and behaviours concerning mpox from an online survey among MSM in the Eastern Mediterranean Region (EMR)
Source: PLoS One. 2026 Nov 13;21(11):e0360183. doi: 10.1371/journal.pone.0360183 (PMC13766248; doi:10.1371/journal.pone.0360183)
Supplement: Supplementary file 2 [file pone.0360183.s002.docx]

**Knowledge, attitude, and behaviours concerning mpox from an online survey among MSM in the Eastern Mediterranean Region (EMR)**

Table S1. List of the NGOs that aided in data collection

| **Name** | **Country** | **List of Services Provided** |
| --- | --- | --- |
| **NGO 1** | Tunisia | Provides victims of discrimination with safe spaces for support and psychological follow-up. It suggests locations for peer listening among community members and offers details on the legal rights of LGBTQI+ individuals |
| **NGO 2** | Morocco | Provides HIV services (Testing, counselling and support for people living with HIV) |
| **NGO 3** | Tunisia | Provides support including medical services for key populations (LGBTI, sex workers, etc.) It deals with detoxification, drug rehabilitation and psychosocial support for people living with HIV diagnosed during their hospitalisation. |
| **NGO 4** | Tunisia | Areas of intervention: 1- Prevention and promotion of risk minimisation behaviours among HIV and key populations 2- Assistance in the psychosocial, legal and medical care of HIV and key populations 3- Advocacy with elected officials, deputies and political institutions for the change of laws and the preservation of the dignity and individual freedoms of HIV and key populations. |
| **NGO 5** | Algeria | Provides HIV Services for people living with HIV |
| **NGO 6** | Algeria | Provides HIV Services for people living with HIV |
| **NGO 7** | Lebanon | **1- Sexual & Reproductive Health & Rights** Clinical Services: Anonymous HIV/Hepatitis B&C and Syphilis voluntary testing and counselling. Medical specialist consultations: STI testing, treatment and follow up. Gynaecological & Midwife consultations and follow-up Other services including vaccines, pap smear, blood tests. Distribution of preventive materials & kits. Referral **2-Human and Gender Rights:** Documentation of human rights violations and abuse. Offering legal support. Referring and reporting cases to other human rights organisations. Advocacy and stigma & discrimination elimination campaigns. Research, policy development and law reform. **3-Quality Care Services for PLHIV** **4-Harm Reduction Services** **5- Mental Health** Scale-up evidence-based and multidisciplinary approaches to Mental Health and substance use for youth and targeted populations (Prevention, treatment and promotion) Psycho-education and awareness  Scaling-up innovative mental health prevention interventions targeting vulnerable populations and vulnerable adolescents and youth to prevent harmful substance use and other risky behaviours Ensure Culturally-adapted standards for mental healthcare and support for the vulnerable populations  **6-Meaningful Engagement**  Empower vulnerable populations to lead and drive their own solutions and reach their potential Ensure economic stimulus packages social connectedness Increase diversity, inclusion and participation of vulnerable populations in governance and civic engagement Ensure agile and flexible services adapting to emergencies and pandemic situations with focus on social protection schemes and support Introducing activities and services that cover the basic livelihoods needs: cash for protection, cash to pay rent and food and provide referrals (Social protection) Develop preparedness and risk plan (test and treatment) to mitigate emerging issues and ensure that services are not interrupted |
| **NGO 8** | Lebanon | **The Voluntary Counselling and Testing (VCT)** VCT is an ongoing activity whereby the National AIDS Program team trains healthcare workers and nurses from various NGO’s, health centres and clinics on VCT service delivery. This activity is done, with the support of UN agencies including WHO and UNICEF. The workshops help to develop new capacities for VCT services, increasing the availability and spread of VCT centres all around Lebanon. The service offers: Information and brochures about HIV/AIDS and STIs; Pre- test counselling, testing and post-test counselling in a private anonymous setting; and Referral to the needed healthcare and social services. **Center for the Distribution of HIV/AIDS Medications** As a step towards reducing stigma and discrimination associated with People living with HIV/AIDS, the NAP opened a centre for the distribution of HIV/AIDS medications; a centre that is independent of the Ministry’s dispensary. The NAP dispensary team always provides a continuous follow up with the drug companies to ensure continuous sustainability and availability of treatment for people living with HIV/AIDS **Drop-in Center (DIC)** In coordination with UNODC and specialised NGO’s like SIDC, SKOUN and AJEM, the NAP opened a drop-in centre (DIC) to host people who use drugs especially injecting and other high-risk groups. The DIC is a place where beneficiaries feel comfortable and are given awareness and education sessions. Also, they can use the VCT facility that exists in the DIC and later on if needed, can be referred to more specialised services. This DIC is a pioneer in the region and Lebanon and plans are being put to later open in other geographical area in Lebanon.  **Awareness Sessions** NAP is constantly raising awareness lectures to the young generation through lectures in schools and universities. |
| **NGO 9** | Lebanon | **1- Legal Support** Proud Lebanon provides legal support and representation to the all beneficiaries despite their nationality or sexual orientation (including Syrian, Iraqi and Palestinian refugees) LGBTIQ+ individuals and prisoners (cases will be selected based on set vulnerability criteria) **2-Free-of-charge rapid HIV and other STI tests** **3- Psycho-social Support** **4- Empowerment Activities** |
| **NGO 10** | Pakistan | Humraz targets males who have sex with males and their partners by providing services which include volunteer counselling and testing, sexually transmitted infection diagnosis and treatment, psychosocial counselling, distribution of condoms  and water-based lubricant as well as advocating for sexual,  welfare and human rights. |
| **NGO 11** | Jordan | Offers: **Outreach** FOCCEC carries out the outreach program via social media platforms and field visits conducted by outreach workers. The outreach program aims to raise the target communities' SRHR awareness and make the SRHR knowledge resources and services accessible to people living with HIV and key populations in Jordan. **Voluntary Counselling and Testing**  The VCT service is free and aims to address beneficiaries' concerns about HIV and STIs through voluntary testing and counselling**.** **Psychosocial Support** **Hotline** FOCCEC has a hotline handled by specialists who answer calls about various concerns related to HIV/STIs and other SRHR issues.  **Case Management** FOCCEC listens to the beneficiaries, identifies their problems, and works with them to design customised plans to help them deal with the challenges that they encounter in accessing SRHR services. **Cash Assistance for Protection** **Follow-up and Aftercare** **Home Visits** **Legal Consultation**  **REAct**  FOCCEC trains outreach workers and counsellors in the field to observe and support individuals who are victims of human rights violations. FOCCEC is focused on supporting these individuals, reporting human rights violations due as a member of national coalitions, and advocating for changes in policy to prevent future gender-based violence issues. **Safe Space** The safe space service provides a relaxing and comfortable place to all genders, ages, and nationalities in Jordan. During opening hours, clients have access to FOCCEC’s facilities such as the TV, running machine, and free Wi-Fi.  **Medical Consultants** **Referral Services** **Media Awareness** |
| **NGO 12** | Egypt | Al Shehab offers three distinct services:  **Voluntary Counselling and Testing (VCT)** addressing the growing importance of HIV/AIDS prevention, care, and support.  **Psychological services** to the target groups (Women, youth, and children) who experienced physical and/or expose to GBV.  **Lega**l **support** includes different services such as legal education and consultation, support to issue official documents and representation in lawsuits. |
