# Supplementary material for: Knowledge, attitude, and behaviours concerning mpox from an online survey among MSM in the Eastern Mediterranean Region (EMR)
Source: PLoS One. 2026 Nov 13;21(11):e0360183. doi: 10.1371/journal.pone.0360183 (PMC13766248; doi:10.1371/journal.pone.0360183)
Supplement: Supplementary file 3 [file pone.0360183.s003.docx]

**Knowledge, attitude, and behaviours concerning mpox from an online survey among MSM in the Eastern Mediterranean Region (EMR)**

Table S2. List of the dating applications and LGBTQ+ websites the mpox survey was advertised on

| **Dating application names** | **LGBTQ+ website names** |
| --- | --- |
| Grindr | thepinknews.com |
| Hornet | out.com |
| Blued: Gay Live Chat and Dating | sdlgbtn.com |
| Growlr | lgbtqnation.com |
| Shuggr-Gay Chat and Dating | towleroad.com |
| Tinder |  |
| Adam4Adam |  |
| Manhunt |  |
| Boyahoy |  |
| Wapo |  |
|  |  |
