# Supplementary material for: Knowledge, attitude, and behaviours concerning mpox from an online survey among MSM in the Eastern Mediterranean Region (EMR)
Source: PLoS One. 2026 Nov 13;21(11):e0360183. doi: 10.1371/journal.pone.0360183 (PMC13766248; doi:10.1371/journal.pone.0360183)
Supplement: Supplementary file 4 [file pone.0360183.s004.docx]

**Knowledge, attitude, and behaviours concerning mpox from an online survey among MSM in the Eastern Mediterranean Region (EMR)**

Table S3. Access to healthcare services and stigma related to mpox by knowledge among study participants

|  | **Total** | | **Low (score≤12)** | | **High (score>12)** | | | **OR** | **(95% CI)** |
| --- | --- | --- | --- | --- | --- | --- | --- | --- | --- |
|  | **n** | **(%)** | **n=512** | **(79.63%)** | **n=131** | | **(20.37%)** |  |  |
| **ACCESS TO CARE** |  |  |  |  |  |  | |  | |
| **Respondent knows where to receive the mpox vaccine** | | | | | | | | | |
| No | 333 | (51.80) | 273 | (53.30) | 60 | (45.80) | | 1 |  |
| Yes | 42 | (6.50) | 32 | (6.20) | 10 | (7.60) | | 1.42 | (0.66-3.04) |
| I don't know | 242 | (37.60) | 187 | (36.50) | 55 | (42.00) | | 1.33 | (0.88-2.01) |
| Prefer not to say | 26 | (4.00) | 20 | (3.90) | 6 | (4.60) | | 1.36 | (0.52-3.54) |
| **Willing to get mpox vaccine** |  |  |  |  |  |  | |  |  |
| No | 73 | (11.40) | 49 | (9.60) | 24 | (18.30) | | 1 |  |
| Yes | 477 | (74.20) | 392 | (76.60) | 85 | (64.90) | | **0.44** | **(0.25-0.76)** |
| Unsure | 93 | (14.50) | 71 | (13.90) | 22 | (16.80) | | 0.63 | (0.31-1.25) |
| **Willing to pay for mpox vaccine** |  |  |  |  |  |  | |  |  |
| No | 246 | (38.26) | 201 | (39.30) | 45 | (34.40) | | 1 |  |
| Yes | 260 | (40.44) | 196 | (38.30) | 64 | (48.90) | | 1.46 | (0.95-2.24) |
| I don’t know | 137 | (21.31) | 115 | (22.50) | 22 | (16.80) | | 0.85 | (0.49-1.49) |
| **Sources of healthcare information** |  |  |  |  |  |  | |  |  |
| **Discussion with family and friends** |  |  |  |  |  |  | |  |  |
| No | 452 | (70.30) | 375 | (73.20) | 77 | (58.80) | | 1 |  |
| Yes | 191 | (29.70) | 137 | (26.80) | 54 | (41.20) | | **1.92** | **(1.29-2.86)** |
| **TV** |  |  |  |  |  |  | |  |  |
| No | 468 | (72.78) | 388 | (75.80) | 80 | (61.10) | | 1 |  |
| Yes | 175 | (27.22) | 124 | (24.20) | 51 | (38.90) | | **1.99** | **(1.33-2.99)** |
| **Healthcare provider** |  |  |  |  |  |  | |  |  |
| No | 208 | (32.35) | 175 | (34.20) | 33 | (25.20) | | 1 |  |
| Yes | 435 | (67.65) | 337 | (65.80) | 98 | (74.80) | | 1.54 | (1.00-2.38) |
| **School or university** |  |  |  |  |  |  | |  |  |
| No | 587 | (91.29) | 462 | (90.20) | 125 | (95.40) | | 1 |  |
| Yes | 56 | (8.71) | 50 | (9.80) | 6 | (4.60) | | 0.44 | (0.19-1.06) |
| **NGO** |  |  |  |  |  |  | |  |  |
| No | 437 | (67.96) | 342 | (66.80) | 95 | (72.50) | | 1 |  |
| Yes | 206 | (32.04) | 170 | (33.20) | 36 | (27.50) | | 0.76 | (0.50-1.17) |
| **FEAR/ STIGMA** |  |  |  |  |  |  | |  |  |
| **Primary reason preventing from receiving the mpox test** | | |  |  |  |  | |  |  |
| Afraid of legal issues | 73 | (11.40) | 42 | (8.20) | 31 | (23.70) | | 1 |  |
| Cost of the test | 123 | (19.10) | 93 | (18.20) | 30 | (22.90) | | **0.43** | **(0.23-0.81)** |
| Don’t know where to get tested | 326 | (50.70) | 274 | (53.50) | 52 | (39.70) | | **0.25** | **(0.14-0.44)** |
| I would be worried about others finding out I got tested | 58 | (9.00) | 49 | (9.60) | 9 | (6.90) | | **0.24** | **(0.10-0.58)** |
| Stigma and discrimination | 63 | (9.80) | 54 | (10.50) | 9 | (6.90) | | **0.22** | **(0.09-0.52)** |
| **Willing to share details of last sexual encounter** | |  |  |  |  |  | |  |  |
| No | 161 | (25.00) | 121 | (23.60) | 40 | (30.50) | | 1 |  |
| Yes | 300 | (46.70) | 257 | (50.20) | 43 | (32.80) | | **0.51** | **(0.31-0.81)** |
| I don't know | 147 | (22.90) | 102 | (19.90) | 45 | (34.40) | | 1.33 | (0.81-2.21) |
| Prefer not to say | 35 | (5.40) | 32 | (6.20) | 3 | (2.30) | | **0.28** | **(0.08-0.97)** |
