# Supplementary material for: Knowledge, attitude, and behaviours concerning mpox from an online survey among MSM in the Eastern Mediterranean Region (EMR)
Source: PLoS One. 2026 Nov 13;21(11):e0360183. doi: 10.1371/journal.pone.0360183 (PMC13766248; doi:10.1371/journal.pone.0360183)
Supplement: Supplementary file 6 [file pone.0360183.s006.docx]

**Knowledge, attitude, and behaviours concerning mpox from an online survey among MSM in the Eastern Mediterranean Region (EMR)**

Figure S2: LASSO Adaptive optimized-adjusted model performance
